# Massive Allograft Reconstruction in Limb Salvage Surgery: Insights From a Single-Institution Case Series in Malaysia

**DOI:** 10.7759/cureus.85217

**Published:** 2025-06-02

**Authors:** Sanjeevan R, Wan Yuhana W Md Yusof, Ang JS, Prashant N, Azuhairy A

**Affiliations:** 1 Orthopedics and Traumatology, Penang General Hospital, George Town, MYS; 2 Orthopedics and Traumatology, Penang Adventist Hospital, George Town, MYS

**Keywords:** allograft union and healing, bone allograft complications, intercalary bone allograft outcomes, limb salvage surgery, massive bone allograft, osteoarticular bone allograft outcomes

## Abstract

Introduction

With advances in imaging and oncological treatment, limb salvage surgery has become a preferred option in musculoskeletal oncology. As survival rates improve, reconstruction durability becomes critical. Massive bone allografts offer a biological solution that preserves bone stock and joint integrity, but they are associated with notable complications. This series aims to evaluate the clinical outcomes of massive allograft reconstructions following tumor resections.

Methods

This is an observational and descriptive review of limb salvage surgeries performed at Penang General Hospital, Malaysia, between 2016 and 2023. It includes a total of 17 patients, comprising 16 individuals diagnosed with primary bone tumors, either malignant or benign but locally aggressive, and one patient who underwent revision surgery for aseptic loosening of a previously implanted prosthesis. All reconstructions were performed using fresh-frozen massive allografts (12 osteoarticular, 5 intercalary) sourced from the Universiti Malaya Medical Centre Bone Bank. Data were collected from medical records, radiographs, and follow-up visits, with limb function evaluated using the Musculoskeletal Tumor Society (MSTS) score. Follow-up ranged from 18 months to nine years.

Results

The cohort included 13 males and four females, with a mean age of 26.7 years (range: 7-71). Histological diagnoses were osteosarcoma (n = 9), Ewing’s sarcoma (n = 3), chondrosarcoma (n = 3), and one giant cell tumor. The patient survival rate was 88.2% (n = 15) at a median follow-up of seven years (range: 3-9 years). Allograft union occurred in 12 cases (70.6%) with a mean time to union of 10.4 months. Four patients developed nonunion, three underwent revision surgery, and one was managed conservatively. Fractures occurred in three patients (17.6%), all requiring osteosynthesis. Infections were reported in three cases, all managed surgically; one case had a delayed onset at 50 months postoperatively. Local recurrence was seen in one patient, necessitating amputation. Seven patients (41.2%) required secondary surgery for complications. Allograft survival rate was 70.6%, and 53% of patients remained complication-free at final follow-up. The mean MSTS functional score (excluding two deceased patients) was 24.

Conclusion

Massive bone allografts remain a viable and durable option for reconstruction in limb salvage surgery following musculoskeletal tumor resection. Despite a 41.2% reoperation rate due to complications such as nonunion, fracture, and infection, long-term outcomes were acceptable, with satisfactory function achieved in the majority of patients. This method supports preservation of joint structures and bone stock, making it particularly beneficial for young patients with extended survival expectations. Careful patient selection and surgical planning are critical to optimize outcomes.

## Introduction

Advancements in imaging and treatment have markedly elevated oncological outcomes in limb salvage surgery, leading to improved survival rates and extended life expectancy. Nevertheless, this success brings forth substantial challenges in achieving reconstructions that are both durable and enduring, while minimizing the risk of complications. Given the concerns surrounding the durability of prosthetic materials and the rising survivorship of sarcoma patients, greater focus has shifted toward biologic reconstructive alternatives [1].

Massive bone allograft is a useful armamentarium in the practice of orthopedic oncology. Although they are associated with various complications, such as infection, fracture, and nonunion, and negative effects on the strength and elastic modulus of the graft due to processing techniques [2-5], the outcome can be very good in selected patients. The decision on the best reconstruction approach for each patient, based on its pros and cons, is ultimately at the discretion of the clinician. For decades, large-segment allografts have served as a longstanding biological solution for repairing bone defects in clinical settings.

This review aims to present a series of patients with musculoskeletal tumors who underwent limb salvage surgery, utilizing fresh frozen bone allografts for reconstruction. The primary objective was to evaluate the outcomes of these allografts in major reconstructive procedures following tumor resection, with a focus on bone union, complications, and functional outcomes.

## Materials and methods

This is an observational and descriptive series of limb salvage surgery with allograft reconstruction in patients with musculoskeletal tumors, conducted at Penang General Hospital between 2016 and 2023. The series includes 17 patients: 16 diagnosed with primary bone tumors, either malignant or benign but locally aggressive, and one patient who underwent revision surgery for aseptic loosening of a previously implanted prosthesis. All patients underwent limb salvage procedures involving massive bone allografts, which included both intercalary and osteoarticular graft types. The allografts were obtained from the bone bank at Universiti Malaya Medical Centre (UMMC), where they were processed using standard protocols that include deep freezing at -80°C following thorough donor screening and tissue harvesting under sterile conditions. The grafts were preserved and stored in sterile packaging and underwent quality control checks in accordance with the bone bank’s guidelines.

Patients who were lost to follow-up within six months postoperatively were excluded from the series to ensure adequate assessment of early and mid-term outcomes. The duration of follow-up for the included patients ranged from 18 months to nine years. Functional evaluation of the limb was conducted using validated scoring systems from the International Society of Limb Salvage (ISOLS) and the Musculoskeletal Tumor Society (MSTS), based on clinical assessment, patient-reported outcomes, and radiographic analysis. All relevant data were retrospectively collected from hospital medical records, radiographs, and outpatient follow-up visits. As this is a retrospective case series, no statistical analysis was performed.

## Results

Demographic data

**Figure 1:**
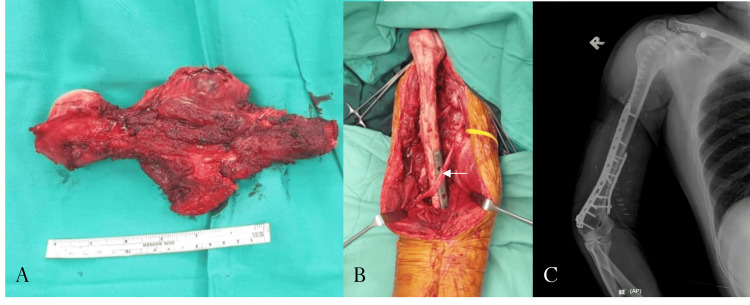
Wide resection of proximal humerus Ewing sarcoma and reconstruction with osteoarticular proximal humerus allograft (A) Specimen of the resected proximal humerus. (B) Postplate fixation of the osteoarticular allograft; the radial nerve is seen transposed anteriorly (white arrow). (C) Postoperative radiograph

**Figure 2:**
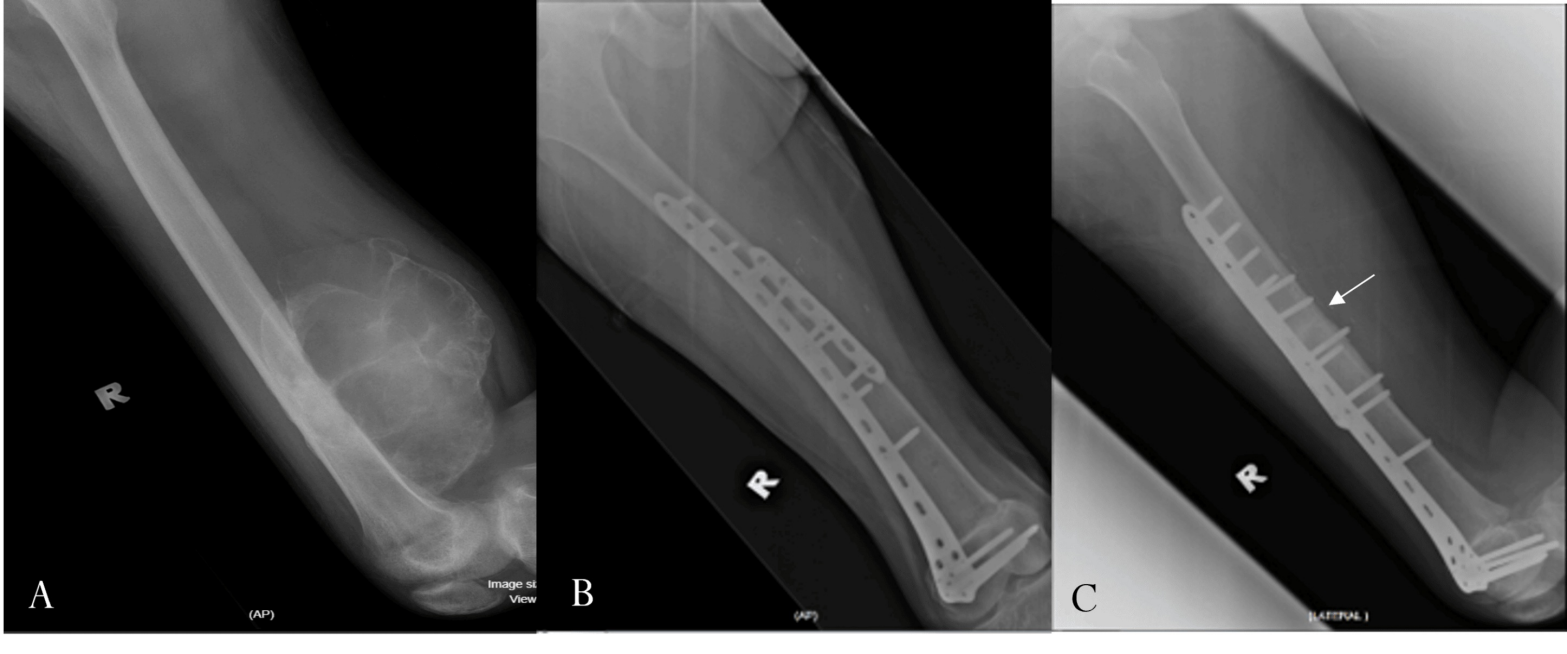
Radiographs of a distal femur osteosarcoma which was resected and reconstructed with intercalary allograft (A) Preoperative lateral view radiograph postadjuvant chemotherapy. (B, C) Anteroposterior and lateral view femur radiographs at one year postsurgery showing radiological union (white arrow)

Seventeen patients were included in this series, ranging in age from seven to 71 years (mean: 26.7 years), comprising 13 males and four females. Of these, 16 underwent primary reconstruction following oncological resection. The histopathological diagnoses included osteosarcoma (n = 9), Ewing’s sarcoma (n = 3), chondrosarcoma (n = 3), and one case of a giant cell tumor involving the distal tibia. One patient, an osteosarcoma survivor, underwent revision surgery for aseptic loosening of a proximal humerus endoprosthesis. In total, 17 allografts were used across the cohort, including 12 osteoarticular grafts (Figure 1) and five intercalary grafts (Figure 2). 

Survival

Two patients, both diagnosed with Ewing’s sarcoma, succumbed to disease progression at 18 months and 24 months postoperatively, respectively. The overall patient survival rate in this series was 88.2%.

Union

**Figure 3:**
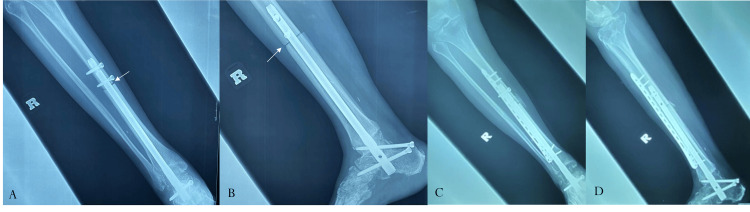
Radiographs of a distal tibia osteosarcoma postresection and reconstruction with an allograft and hindfoot arthrodesis nail (A, B) Host-donor junction with no evidence of union (white arrows) due to construct instability (at eight months postsurgery). (C,D) Postplating and corticocancellous bone grafting at the host-donor junction to facilitate union

Of the 17 cases, 12 achieved union at the host-donor junction, with an average time to union of 10.4 months. Notably, one case required 24 months for union due to instability at the host-donor junction. This was eventually stabilized with a plate, and a corticocancellous graft was inserted to facilitate union (Figure 3).

Nonunion at the host-donor junction was observed in four cases, involving three femoral and one tibial reconstruction. Among these, one patient sustained a fracture and underwent surgery prior to succumbing to pulmonary metastasis; two developed concurrent infections and required reoperation; and one case was managed nonoperatively.

Infection

Infective complications were observed in three cases (17.6%), with onset ranging from two to 50 months postoperatively. All cases required surgical intervention. Two patients underwent staged procedures consisting of debridement, graft removal, and insertion of an antibiotic-loaded cement spacer. One of these patients subsequently received a distal femur endoprosthesis, while the other underwent bone transport with successful ankle arthrodesis, despite the presence of a persistent discharging sinus at the ankle.

The third patient, who developed a delayed infection at 50 months postoperatively, was managed with multiple debridements, plate removal, graft retention, and prolonged antibiotic therapy until normalization of inflammatory markers. This patient had a fracture the following year and underwent osteosynthesis and bone grafting.

Fracture

Allograft fractures were observed in three patients (17.6%), all of whom were managed with osteosynthesis. Two of these patients survived, while one unfortunately succumbed to disease progression.

Recurrence

One patient with parosteal osteosarcoma of the distal femur underwent amputation following local recurrence at 15 months after the index surgery. The patient has stable lung nodules, developed multiple soft tissue metastases over the scalp and contralateral thigh over the years, and is still functional and surviving to date.

Reoperation and allograft survival

Secondary surgical interventions were required in seven patients, with indications including local recurrence, infection, nonunion, delayed union, and allograft fracture, resulting in a reoperation rate of 41.2%. Accounting for graft removal, patient deaths, and amputation, the overall allograft survival rate was 70.6%. At the latest follow-up, 53% of our patients (n = 9) experienced no major complications and did not require any reoperation.

Functional score

The MSTS score at the latest follow-up after the reoperation was used as the final functional score. Excluding the two deceased patients, the mean MSTS functional score among the remaining patients was 24.

## Discussion

Allograft offers a biological reconstruction option for large skeletal defects, aiding to reestablish skeletal continuity and function after bone tumor resection [4,6]. They provide structural support and can bear mechanical loads [4,6], even before significant revascularization and creeping substitution occur [7]. A key advantage of massive allografts is their ability to support anatomical reattachment of host tendons, ligaments, and muscles, which could contribute to better functional outcomes [4,6]. Preservation of the bone stock is one of the valued benefits of allografts, offering an advantage for potential future procedures, particularly when host bone available for fixation is limited [6,8]. Massive bone allograft reconstruction carries a significant risk of complications, underscoring the need for careful patient selection and surgical planning. The most commonly encountered major complications include nonunion, graft fracture, and deep infection [2,5,8-10].

Healing at the allograft-host bone interface is typically characterized by a slow and prolonged process [6,10,11]. Our series demonstrated a union rate of 70.6% (n = 12) with an average time to achieve union being 10.4 months. Cortical host-donor junction has been reported to heal between eight and 15 months [10]. In a study by Liu et al. involving 38 patients and 70 host-donor junctions, complete union was achieved in 30 junctions, with a mean time to union of 13 months [2]. We noted one case with delayed union, which took 24 months and required additional surgical stabilization and autografting to unite. It has been highlighted that robust internal fixation is a crucial element for ensuring stability at the osteotomy surface and achieving allograft survival, which helps avoid nonunion due to micromovement [2,4].

Combining an intercalary allograft with a vascularized fibular graft may accelerate osseous union at the osteotomy sites. Capanna et al. cited an overall success rate for this combined technique of 93.5% [12]. They compare this to rates of 80% with allograft alone and 85% with vascularized fibula alone [12]. Furthermore, in pediatric cases, potential growth may be achieved by implanting a vascularized fibula with its growth plate preserved [12].

Factors that can influence the time to union include the security of fixation and the degree of contact at the junction [6], the use of autogenous bone graft at the junction to promote callus formation [4,6,10] and potentially the type of osteotomy (e.g., V-shaped or stepped) to increase contact surface and stability [2]. One study found that adjuvant chemotherapy increased the incidence of delayed union, although it was not linked to a higher risk of fracture or infection [11]. In contrast, another study reported no significant effect on the time to union [10].

Infection is widely recognized as a major complication following massive bone allograft reconstruction, which often leads to the failure of the allograft [9]. The anatomical site influences infection risk, with tibial reconstructions exhibiting higher susceptibility due to limited soft tissue coverage [8,12]. Another risk factor for infection was adjuvant therapy, especially when both chemotherapy and radiation were used [12]. Patients receiving chemotherapy were more than four times as likely to experience complications, including infection [8]. Radiation alone or combined with chemotherapy also significantly increased infection rates compared to no adjunctive therapy [9]. Chemotherapy may delay wound healing and lead to infection [11].

Reoperations for complications such as nonunion or fracture are associated with a significantly increased risk of infection, with a higher number of reoperations observed among patients who developed postoperative infections [9]. In one of our patients, a delayed infection was identified 50 months after the index procedure. Notably, the patient had undergone a limb lengthening surgery on the same limb seven months prior, which may have contributed to the onset of infection. This suggests that secondary surgical interventions, even remote from the primary reconstruction, may act as potential risk factors for late-onset infection in allograft recipients. Blood transfusion has been identified as a risk factor for postoperative infection, with notably higher infection rates observed in patients receiving more than 1000 mL [13].

Although recurrence rates vary across long-term studies, key reports provide useful data. Mankin et al. [5] reported a 10% recurrence rate in high-grade tumors over 72 months of follow-up. Ippolito et al. [8] observed five recurrences (6.8%) in 74 patients over 105 months. Both studies highlight local recurrence as a significant factor impacting long-term outcomes of allograft reconstruction.

The average MSTS score in our patient cohort was 24, which is comparable to the findings of a study evaluating patients with complications after allograft reconstruction, where the mean score was reported as 23.6 [8]. Ippolito et al in a similar study concluded that despite considerable complication rates, managing these complications with a systematic approach resulted in successful outcomes [8]. A study focusing on massive bone allograft reconstruction in patients with high-grade osteosarcoma reported that functional results were satisfactory (excellent or good) in 74% of patients after the primary surgery and improved to 83% after secondary surgery [11].

This case series supports the use of massive allografts as a durable and effective option for limb reconstruction following tumor resection, with an overall patient survival rate of 88.2% and allograft survival of 70.6%. Despite a 41.2% reoperation rate due to complications such as infection, nonunion, and fracture, 53% of patients remained complication-free at the latest follow-up. The mean MSTS functional score of 24 reflects satisfactory functional outcomes, supporting the role of allografts as a durable and effective reconstructive method in oncologic limb salvage surgery.

As this series involves a small number of patients, the results may not fully reflect the range of potential outcomes in a wider population. Expanding this to a multicenter study with a larger cohort would provide more definitive conclusions.

## Conclusions

Reconstruction with massive allografts continues to be a valuable approach, offering long-term structural integrity despite the risk of complications. It remains a cost-efficient strategy that allows for preservation of the native joint when feasible or can be integrated with endoprosthetic components to limit the extent of metal implantation. Additionally, it supports the conservation of bone stock, which is increasingly important as sarcoma patients experience longer survival and may require future revision procedures.
